# Global MicroRNAs Expression Profile Analysis Reveals Possible Regulatory Mechanisms of Brain Injury Induced by *Toxoplasma gondii* Infection

**DOI:** 10.3389/fnins.2022.827570

**Published:** 2022-03-10

**Authors:** Zhaofeng Hou, Lele Wang, Dingzeyang Su, Weimin Cai, Yu Zhu, Dandan Liu, Siyang Huang, Jinjun Xu, Zhiming Pan, Jianping Tao

**Affiliations:** ^1^College of Veterinary Medicine, Yangzhou University, Yangzhou, China; ^2^Jiangsu Co-innovation Center for Prevention and Control of Important Animal Infectious Diseases and Zoonoses, Yangzhou, China; ^3^Jiangsu Key Laboratory of Zoonosis, Yangzhou, China

**Keywords:** *Toxoplasma gondii*, pig, microRNA, brain injury, acute and chronic infection

## Abstract

*Toxoplasma gondii* (*T. gondii*) is an obligate intracellular parasitic protozoan that can cause toxoplasmosis in humans and other endotherms. *T. gondii* can manipulate the host gene expression profile by interfering with miRNA expression, which is closely associated with the molecular mechanisms of *T. gondii*-induced brain injury. However, it is unclear how *T. gondii* manipulates the gene expression of central nervous system (CNS) cells through modulation of miRNA expression *in vivo* during acute and chronic infection. Therefore, high-throughput sequencing was used to investigate expression profiles of brain miRNAs at 10, 25, and 50 days post-infection (DPI) in pigs infected with the Chinese I genotype *T. gondii* strain in this study. Compared with the control group 87, 68, and 135 differentially expressed miRNAs (DEMs) were identified in the infected porcine brains at 10, 25, and 50 DPI, respectively. Gene Ontology (GO) and Kyoto Encyclopedia of Genes and Genomes (KEGG) pathway enrichment analysis showed that a large number significantly enriched GO terms and KEGG pathways were found, and were mostly associated with stimulus or immune response, signal transduction, cell death or apoptosis, metabolic processes, immune system or diseases, and cancers. miRNA–gene network analysis revealed that the crucial connecting nodes, including DEMs and their target genes, might have key roles in the interactions between porcine brain and *T. gondii*. These results suggest that the regulatory strategies of *T. gondii* are involved in the modulation of a variety of host cell signaling pathways and cellular processes, containing unfolded protein response (UPR), oxidative stress (OS), autophagy, apoptosis, tumorigenesis, and inflammatory responses, by interfering with the global miRNA expression profile of CNS cells, allowing parasites to persist in the host CNS cells and contribute to pathological damage of porcine brain. To our knowledge, this is the first report on miRNA expression profile in porcine brains during acute and chronic *T. gondii* infection *in vivo*. Our results provide new insights into the mechanisms underlying *T. gondii*-induced brain injury during different infection stages and novel targets for developing therapeutic agents against *T. gondii*.

## Introduction

*Toxoplasma gondii* is an obligate intracellular parasitic protozoan that can cause toxoplasmosis in all endotherms, including humans. In immunocompetent patients, *T. gondii* generally causes a chronic, cryptic, latent brain infection that is clinically asymptomatic (Dubey et al., 2012; Wang T. et al., 2019). *T. gondii* in the brain can also modulate the behavior and cognitive function of intermediate hosts (Flegr et al., 2003), and is associated with increased rates of psychiatric disorders and suicide, and decreased psychomotor performance (Torrey and Yolken, 2003; Carruthers and Suzuki, 2007). *T. gondii* infection can also impair learning and memory in rodents (Kannan et al., 2010), and is involved in neurodegenerative disorders in humans, including Alzheimer’s, Huntington, and Parkinson’s diseases (Miman et al., 2010; Kusbeci et al., 2011; Donley et al., 2016). Such evidence suggests that *T. gondii*-induced injury of the cells of the central nervous system (CNS) might have important roles in inducing the occurrence of other neuronal disorders.

As a class of endogenous gene post-transcriptional regulators, microRNAs (miRNAs) mediate the regulation of almost all cell processes, and have been recognized as key molecules to understand the mechanism of disease development and to find disease diagnosis biomarkers and treatment targets. miRNAs are abundant in the CNS and are essential in the regulation of neuronal development and function (Tang and Sun, 2020). *T. gondii* is able to manipulate the global gene expression profiles of host cell by interfering with miRNA expression of the invaded cells (Hakimi and Cannella, 2011). miRNAs involved in host brain responses to *T. gondii* infection are important regulators of early parasite dissemination in the brain tissue (Cannella et al., 2014). The invasion of cyst-forming *T. gondii* can change the miRNA expression of host brain, to which the host attempts to respond using two tactics: marking proteins with “protein tags” and adaptation of immune-related systems (Xu et al., 2013).

The distribution of *T. gondii* genotypes varies worldwide. However, type Chinese I (ToxoDB #9) is the predominant genotype of *T. gondii* prevalent in both humans and animals in China (Wang et al., 2013). miRNA expression alterations of the murine brain harboring *T. gondii* have been documented (Xu et al., 2013; Hu et al., 2018; Zhou et al., 2020), but less is known about how Chinese I genotype strain manipulates gene expression in CNS cells during acute and chronic infection in endotherms, including pigs. *T. gondii* is one of the major causes of abortion and stillbirth in pigs. Acute *T. gondii* infection can induce onset of toxoplasmosis and death in pigs, whereas, during chronic infection, tissue cysts remaining in the brain or muscles of pigs can damage public health (Schlüter et al., 2014). In addition, a growing number of studies showed that a common molecular mechanism inducing brain injury may exist between neurodegenerative disorders and toxoplasmosis (Martin, 1999; Hermes et al., 2008; Elsheikha et al., 2016). Thus, as a suitable model organism for comparative genomics and biomedical studies, the roles of porcine brain miRNAs in the occurrence and development of neurological toxoplasmosis will provide a research basis for *T. gondii*-induced neuronal disorders in humans.

In the current study, we performed a high-throughput sequencing analysis to identify differentially expressed miRNAs (DEMs) between infected and non-infected porcine brain samples at different time points to investigate the potential roles of miRNAs in *T. gondii*-induced injury to CNS cells during acute and chronic infections. Therefore, our results provide not only new insights into the mechanisms underlying *T. gondii*-induced neuropathology *in vivo* during different infection stages, but also novel targets for developing therapeutic agents against *T. gondii* and a model to reveal the mechanisms underlying neuronal disorders.

## Materials and Methods

### Animals and Parasite Challenge

All experiments and methods involving to animals were carried out in accordance with the ARRIVE guidelines (Animal Research: Reporting of *in vivo* Experiments) (Percie du Sert et al., 2020) in this study. Twenty-four 5-week-old commercial male pigs that has been castrated, each weighing 7–7.5 kg, were obtained from the Jiangsu Meilin Animal Husbandry Co., Ltd. (Jiangsu, China), and proven to be free from *T. gondii*, classical swine fever virus, porcine reproductive and respiratory syndrome virus, porcine pseudorabies virus, porcine parvovirus, and porcine circovirus virus by specific IgM and IgG antibody tests using ELISA or the whole-blood nucleic acid detection using PCR methods. All animals were housed individually with the same food and water in standard breeding houses for 1 week before infection.

The *T. gondii* YZ-1 strain was isolated from a home-bred wild boar in Jiangsu Province of China in 2011. In a previous study, this strain was identified as ToxoDB PCR-RFLP genotype #9 (Chinese I) and shown to be virulent in ICR mice (Hou et al., 2018). Tachyzoites of the YZ-1 strain used in this study were obtained and purified according to previous work (Hou et al., 2018); they were resuspended in sterile phosphate-buffered saline (PBS, pH 7.4) and used to infect piglets within 2 h of resuspension. Pigs were randomly allocated to a control group (nine pigs) or an infection group (15 pigs). For the infection group, each pig was intravenously injected with 5 × 10^7^ purified tachyzoites of YZ-1 strain in 2 ml of PBS, whereas pigs in the control group were sham-injected with 2 ml of PBS only. In addition, five ICR mice, each weighing 20–22 g, were purchased from the Comparative Medicine Center of Yangzhou University (Yangzhou, China), and then were inoculated intraperitoneally with 200 purified tachyzoites of YZ-1 strain to assess parasite viability. All mice were euthanized under deep anesthesia with sodium pentobarbital (150 mg/kg body weight) after experimentation.

### Clinical Symptoms and Histopathological Examination

After infection, clinical symptoms were observed and rectal temperatures were measured twice daily. At 10, 25, and 50 days post infection (DPI), three pigs from each group were sacrificed under deep anesthesia with sodium pentobarbital (200 mg/kg body weight) by captive bolt for small animals. The brain, heart, liver, spleen, lungs, kidneys, and lymph nodes of the pigs were collected and fixed in 10% buffered formalin (pH 7.2) for more than 24 h. They were then dehydrated using a graded series of alcohol, transparentized by xylene, and embedded in paraffin wax. Sections (5 μm thick) were cut from the paraffin wax blocks and stained with Hematoxylin and Eosin (H&E) for histopathological observation.

### *Toxoplasma gondii* Detection by PCR and Mouse Bioassay

Genomic DNA of brain samples from pigs in the infection and control groups was extracted for *T. gondii* detection using two PCR methods targeting the 529-bp repetitive sequence (AF146527) and *B1* gene (AF179871) as previously described (Homan et al., 2000; Hill et al., 2006). The genomic DNA of the YZ-1 strain was set as the positive control, whereas the tissue DNA of a pig confirmed *T. gondii* infection-free by PCR was used as the negative control for each reaction. The brain tissues of the pigs were homogenized and orally inoculated into healthy ICR mice. All inoculated mice were closely monitored for 30 days. Any parasites in ascites fluid of dead mice were observed by light microscopy, and anti-*T. gondii* antibodies in serum of surviving mice were tested at 30 DPI using the *T. gondii* indirect hemagglutination assay (IHA) diagnostic kit (Lanzhou Veterinary Research Institute, Chinese Academy of Agricultural Sciences, Lanzhou, China) following the manufacturer’s instructions.

### Small RNA Library Preparation and Sequencing

Brain samples collected from the three pigs sacrificed from each group at 10, 25, and 50 DPI, were thoroughly rinsed in PBS, immediately frozen in liquid nitrogen, and then stored at −80°C. Total RNA was extracted with TRIzol Reagent (Invitrogen, Carlsbad, CA, United States) according to the manufacturer’s protocol. RNA purity was checked using the NanoPhotometer^®^ spectrophotometer (IMPLEN, CA, United States). RNA concentration was measured by Qubit^®^ RNA Assay Kit in Qubit^®^ 2.0 Flurometer (Life Technologies, CA, United States). The integrity of the total RNA samples was assessed using the RNA Nano 6000 Assay Kit of the Agilent Bioanalyzer 2100 system (Agilent Technologies, CA, United States). Three individual RNA samples with high RNA integrity values from each group at each of the time points were equally pooled. Thus, a total of six small RNA (sRNA) libraries (BI-10, BI-25, and BI-50 in infection group, and BC-10, BC-25, and BC-50 in the control group), were generated using NEBNext^®^ Multiplex Small RNA Library Prep Set for Illumina^®^ (NEB, Ipswich, MA, United States) following the manufacturer’s recommendations and then sequenced by the Novogene Bioinformatics Institute (Beijing, China) on an Illumina Hiseq 2500 platform (Illumina, San Diego, CA, United States) following the manufacturer’s instructions.

### Bioinformatic Analysis

Bioinformatic analysis of the high-throughput sequencing data was performed according to our previous work (Hou et al., 2019). Briefly, custom perl and python scripts were used to process the raw reads, and clean reads were obtained after removing various reads, including ploy-*N*, reads with 5′ adapter contaminants, reads without 3′ adapter or the insert tag, containing ploy A, T, G, C, and low-quality reads from raw data. Reads with lengths longer than 18 nucleotides (nt) or shorter that 35 nt were then selected from the clean reads to align onto the pig genome (Sscrofa10.2) from Ensembl^1^ by Bowtie without mismatch. After removing tags originating from protein-coding genes, repeat sequences, rRNA, tRNA, snRNA, and snoRNA, porcine miRNAs were identified from the mapped sRNA tags using miRBase^2^, and novel miRNAs were predicted by miREvo and mirdeep2 software. Transcript per million clean tags (TPM) was used to assess the expression levels of miRNAs, and differential expression between groups was analyzed using the DEGseq (2010) R package. *P*-values were corrected to obtain *q*-values using the Benjamini and Hochberg method, and a *q*-value < 0.01 and log_2_(fold change) > 1 were set as the threshold for significantly differential expression by default. The target genes of the miRNAs were predicted using miRanda and RNAhybrid. Gene Ontology (GO) and Kyoto Encyclopedia of Genes and Genomes (KEGG) enrichment analyses were performed to identify the cellular function and molecular pathways regulated by the DEMs (Kanehisa et al., 2017; Young et al., 2010). To gain insight into the interactions between miRNAs and target genes related to brain damage caused by *T. gondii* infection, miRNA–gene networks were created and visualized using Cytoscape 3.4.0 software (Shannon et al., 2003). Full details of the sequence data were submitted to the Gene Expression Omnibus public database (GEO^3^) with the GEO accession number GSE176143. The raw data are available in the NCBI Sequence Read Archive under the accession number SRP322697.

### Validation of MicroRNAs Expression

A SYBR green-based quantitative reverse transcription real-time PCR (RT-qPCR) was used to verify the results of the miRNA expression analyses. Total RNA was extracted from porcine brain samples and reverse transcribed into cDNA by a Mir-x*™* miRNA First-Strand Synthesis and SYBR^®^ RT-qPCR Kit (TaKaRa, Dalian, China) following the manufacturer’s instructions. miRNA sequences and primers were synthesized by BGI Co., Ltd. (Shenzhen, China). The qRT-PCR system and cycling conditions were as per our previous study (Hou et al., 2019). All RT-*q*PCR reactions were performed in triplicate, and snRNA U6 was used as a housekeeping miRNA to normalize and quantify miRNA expression.

## Results

ICR mice inoculated with the purified tachyzoites used for parasite challenge in pigs exhibited the onset of toxoplasmosis at 3 DPI. *T. gondii* tachyzoites were observed in ascites of mice microscopically. These results demonstrated the viability of the parasites used to infect the pigs.

### Clinical Symptoms and Histopathological Examination

**FIGURE 1:**
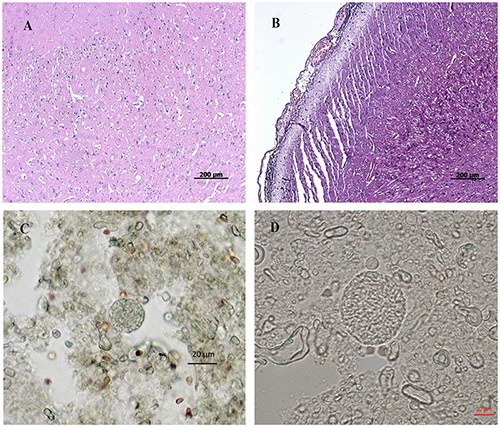
Histopathological observation of pig brain infected by *T. gondii*. **(A**,**B)** represent histopathology of tissue sections prepared from porcine brain by HE staining. **(A)** represents the brain prepared from pigs of the control group. **(B)** represents the brain prepared from pigs in the infected group. **(C,D)** represents tissue cysts of *Toxoplasma* in brain tissue homogenate of the infected pigs. **(A,B)**, bar = 200 μm; **(C)**, bar = 20 μm; **(D)**, bar = 10 μm.

All infected pigs showed characteristic clinical symptoms and principal lesions of *T. gondii* acute infection (Hou et al., 2019). No clinical symptoms were observed in infected pigs from 20 DPI onward. At 50 DPI, the porcine brains showed microscopic lesions, including meningeal inflammation, local vascular dilation, congestion, and inflammatory cell infiltration (Figures 1A,B), whereas porcine lungs showed local interstitial widening and alveoli emphysema. Additionally, tissue cysts were observed in the brain tissues of pigs at 50 DPI (Figures 1C,D), which confirmed that the infected pigs had sustained neuropathological damage. This is the first report of tissue cysts in pigs infected with the Chinese I genotype of *T. gondii*. No clinical symptoms or lesions were observed in the control group at any time point during this study.

### *Toxoplasma gondii* Detection by PCR and Mouse Bioassay

The PCR results showed that brain tissues from the infected pigs at 10, 25, and 50 DPI were *T. gondii* positive, whereas those from the control group were *T. gondii* negative. All the mice inoculated with homogenates of brain tissue from the infected pigs developed toxoplasmosis within 7∼15 days, and numerous *T. gondii* tachyzoites were observed in ascites fluid of these mice. No onset of toxoplasmosis occurred in mice inoculated with the homogenates of brain tissue of control pigs within 30 days, and mouse serum was negative for anti-*T. gondii* antibodies.

### Profile of Sequencing Data

MicroRNA libraries were successfully prepared from the brains of infected and uninfected (control) pigs. In the infected group, 11,387,795, 15,682,714, and 19,448,790 raw reads were obtained from the BI-10, BI-25, and BI-50 sRNA libraries, respectively. In the control group, 20,493,040, 12,082,833, and 12,318,466 raw reads were obtained from the BC-10, BC-25, and BC-50 sRNA libraries, respectively. After removing low-quality reads and masking adaptor sequences, more than 10 million high-quality clean reads were obtained from the six sRNA libraries (Table 1). In the 18∼35 nt sRNAs selected from clean reads, most were 20∼24 nt in length, with most being 22 nt in length. Of the sRNA tags from the six libraries, 83.11–90.68% aligned with the reference pig genome. Based on sequence alignment and category annotation, 4,627,784, 4,738,233, 4,095,744, 1,164,932, 5,691,495, and 6,412,436 total reads were identified as known miRNAs and 993, 1227, 1697, 772, 1164, and 1376 of total reads were predicted to be novel miRNAs from the sRNAs aligned to the reference sequence in the BI-10, BI-25, BI-50, BC-10, BC-25, and BC-50 sRNA libraries, respectively. A total of 313 known miRNAs, corresponding to 300 precursors, was identified with a BLAST search against the miRBase, and 318 novel mature and 75 star miRNAs, corresponding to 324 precursors, were identified by recognition of standard stem-loop structures (Table 2). Of these mature miRNAs, 272 known and 48 novel miRNAs were shared among the six libraries. Twelve of the 20 most abundant miRNAs in each sample (miR-9-1, miR-9, miR-26a, let-7i, miR-181a, miR-128, miR-100, miR-30d, miR-27b-3p, miR-7, miR-127, and miR-103) were co-expressed in all six libraries (Supplementary Table 1). Comparison of the *T. gondii*-infected and uninfected group revealed that 371 miRNAs (286 known and 85 novel) were shared between the BI-10 and BC-10 samples, 404 miRNAs (294 known and 110 novel) between the BI-25 and BC-25 samples, and 394 miRNAs (282 known and 112 novel) between the BI-50 and BC-50 samples (Table 3). By comparison with the control group at the same time point, some miRNAs were found to be infection stage specific, such as miR-1277, miR-202-5p, and miR-7139-5p, detected in BI-10 but not in BC-10, and miR-19a, miR-4332 and miR-758 were identified in BI-50 but not in BC-50 (Supplementary Table 2). However, most of these miRNAs had relatively low expression levels during *T. gondii* infection.

**TABLE 1:** The list of data filtering (%).

Library	Total reads	N% > 10%	Low quality	5′ contamine	3′ null or insert null	With ployA/T/G/C	Clean reads
BI-10	11,387,795 (100)	402 (0.00)	26,589 (0.23)	341 (0.00)	155,345 (1.36)	10,813 (0.09)	11,194,305 (98.30)
BI-25	15,682,714 (100)	122 (0.00)	12,811 (0.08)	1,167 (0.01)	314,296 (2.00)	27,552 (0.18)	15,326,766 (97.73)
BI-50	19,448,790 (100)	74 (0.00)	34,985 (0.18)	835 (0.00)	332,222 (1.71)	32,569 (0.17)	19,048,105 (97.94)
BC-10	20,493,040 (100)	100 (0.00)	33,117 (0.16)	1,626 (0.01)	641,110 (3.13)	86,894 (0.42)	19,730,193 (96.28)
BC-25	12,082,833 (100)	113 (0.00)	20,737 (0.17)	254 (0.00)	144,337 (1.19)	14,304 (0.12)	11,903,088 (98.51)
BC-50	12,318,466 (100)	72 (0.00)	38,869 (0.32)	685 (0.01)	308,249 (2.50)	16,870 (0.14)	11,953,721 (97.04)

**TABLE 2:** The known and novel miRNAs mapped on pig genome.

miRNA	Types	Total	sRNA libraries					
			BI-10	BI-25	BI-50	BC-10	BC-25	BC-50
Known miRNA	Mature	313	301	304	299	287	296	286
	Hairpin	300	280	288	285	284	285	283
	Unique sRNA	26,942	4,628	5,407	4,786	3,769	4,452	3,900
	Total sRNA	26,730,624	4,627,784	4,738,233	4,095,744	1,164,932	5,691,495	6,412,436
Novel miRNA	Mature	318	165	187	192	132	161	163
	Star	75	24	34	36	21	29	31
	Hairpin	324	176	204	214	143	169	175
	Unique sRNA	1,924	301	375	389	243	291	325
	Total sRNA	7,229	993	1,227	1,697	772	1,164	1,376

**TABLE 3:** The comparation of miRNA number between infection and control groups.

Comparations	Common		Infection		Control		Total
	known	novel	known	novel	known	novel	
BI-10 vs BC-10	286	85	15	80	1	47	514
BI-25 vs BC-25	294	110	10	77	3	51	545
BI-50 vs BC-50	282	112	17	80	4	51	546

### Differentially Expressed MicroRNA Analysis

**FIGURE 2:**
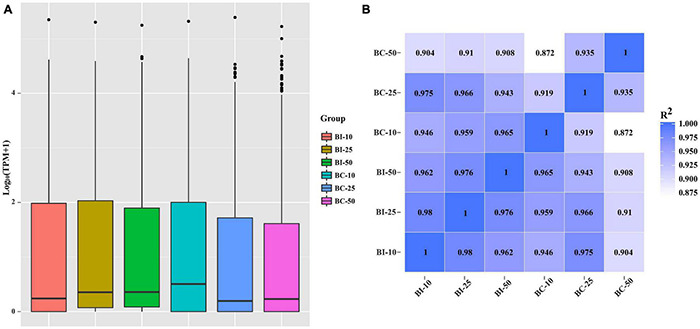
The miRNAs expression distribution and correlation analysis between samples in different groups. **(A)** TPM boxplot of miRNA expression levels in different samples. **(B)** Pearson correlation coefficients were calculated to estimate the association of expression levels between samples.

**FIGURE 3:**
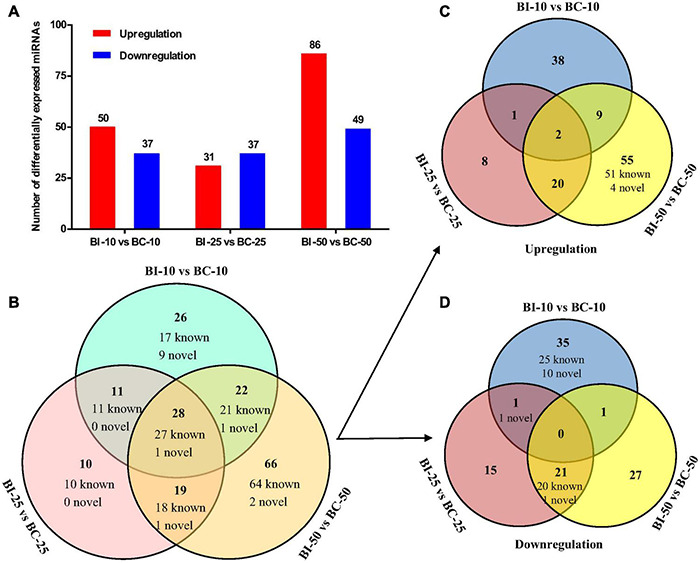
The differential expression analysis of pig brain miRNAs between the infected and control groups. **(A)** The number of DEMs between the infected and control groups at 10, 25, and 50 DPI. **(B**–**D)** The Venn diagrams of the DEMs between the infected and control groups at three time points.

The distribution of miRNA expression in different samples was shown by a TPM boxplot (Figure 2A). Pearson correlation coefficients were used to estimate the expression patterns of miRNAs in different samples, which ranged from 0.872 for BC-10 versus BC-50 to 0.98 for BI-10 versus BI-25 (Figure 2B). In total, 182 unique pig-encoded miRNAs were significantly differentially expressed between the infected and control samples at the three time points (*q* < 0.001), including 14 novel miRNAs. Compared with the control groups at the same time point, 87, 68, and 135 unique miRNAs were identified as DEMs in the BI-10, BI-25, and BI-50 samples, respectively (Figure 3A and Supplementary Tables 3–5). In addition, 102 DEMs were identified to be sample specific, including 26 from BI-10, ten from BI-25, and 66 from BI-50 (Figure 3B). Of 47 DEMs shared by the 25 DPI and 50 DPI time points, the expression patterns of 43 DEMs (91.5%, 43/47) were found to be consistent, including 22 upregulated and 21 downregulated DEMs (Figures 3C,D). Of 39 DEMs shared by the 10 DPI and 25 DPI time points, four DEMs (10.26%, 4/39) had consistent expression patterns. Of 50 DEMs shared by the 10 DPI and 50 DPI time points, 12 DEMs (24.0%, 12/50) had consistent expression patterns (Figures 3C,D). Of the 133 upregulated DEMs, miR-142-3p and miR-142-5p were found in all infected samples (Figure 3C), but none of the shared miRNAs were found in downregulated DEMs in all infected samples (Figure 3D).

### Functional Enrichment Analysis

**FIGURE 4:**
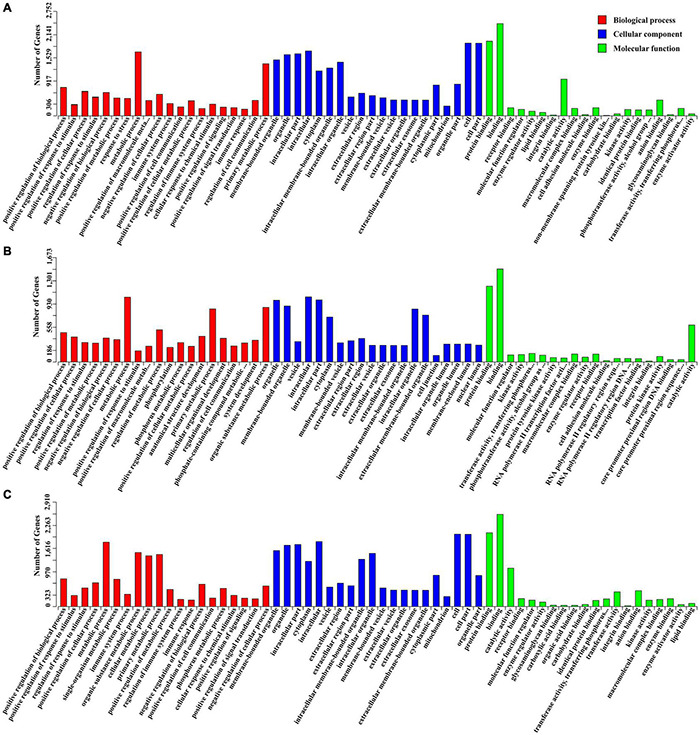
The GO enrichment analysis for target genes of DEMs. **(A)** The significant enriched GO terms of biological process, cellular component and molecular function for target genes of DEMs at 10 DPI. **(B)** The significant enriched GO terms of biological process, cellular component and molecular function for target genes of DEMs at 25 DPI. **(C)** The significant enriched GO terms of biological process, cellular component and molecular function for target genes of DEMs at 50 DPI.

In total, 4277 candidate target genes of DEMs were predicted by RNAhybrid and miRanda software. Of these, 497, 1935 and 3138 target genes were predicted for the upregulated miRNAs, and 3166, 171, and 1158 target genes for the downregulated miRNAs at 10, 25, and 50 DPI, respectively. Based on these putative target genes, GO enrichment analysis was performed to identify the enriched biological processes (BP), cellular components (CC), and molecular functions (MF) of the DEMs, the top 20 of which are shown in Figure 4. Compared with control samples, a total of 534 significantly enriched GO terms (*P* < 0.01) were identified from the infected samples, 191 of which were shared by the BI-10, BI-25, and BI-50 samples, and were mainly related to stimulus or immune response, signal transduction, protein phosphorylation and modification, cell death or apoptosis, metabolic processes, and developmental process. Of 480 GO terms enriched between BI-10 and BC-10, 416, 45, and 19 terms belonged to BP, CC, and MF, respectively (Supplementary Table 6); of 250 GO terms enriched between BI-25 and BC-25, 198, 38, and 14 terms were identified as BP, CC, and MF, respectively (Supplementary Table 6); of 357 GO terms enriched between BI-50 and BC-50, 302, 38, and 17 terms were identified as BP, CC, and MF, respectively (Supplementary Table 6).

**FIGURE 5:**
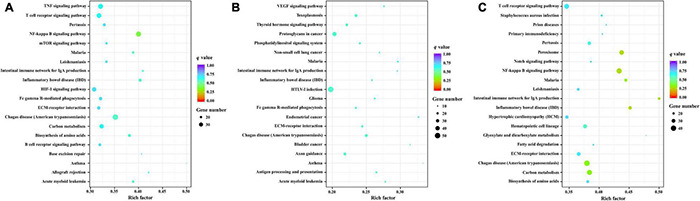
The KEGG enrichment analysis for target genes of DEMs. The top 20 KEGG pathways of differentially expressed miRNAs in porcine brain between the infected and control groups at 10 **(A)**, 25 **(B)**, and 50 **(C)** DPI. Rich factor indicates the ratio of target genes of DEMs enriched in the pathway among genes annotated in the pathway. Adapted from Kanehisa et al. (2017), Young et al. (2010).

Kyoto Encyclopedia of Genes and Genomes enrichment analysis was the performed to identify the roles of the identified DEMs in the porcine brain response to *T. gondii* infection. Compared with control samples, 275 KEGG pathways were enriched in the infected samples. The top 20 KEGG pathways of the target genes of DEMs between infection and control samples at 10, 25 and 50 DPI are shown in Figure 5. KEGG pathways with *P*-values < 0.05 were analyzed further (Supplementary Table 7); the most frequently represented pathways were associated with signal transduction, cancers, immune system or diseases, and infectious diseases containing protozoal diseases, such as malaria (ssc05144), toxoplasmosis (ssc05145), American trypanosomiasis (ssc05142), and leishmaniasis (ssc05140). In addition, 16, 20, and 15 pathways were significantly enriched (*P* < 0.05) at 10, 25, and 50 DPI, respectively. Further analysis found that the pathways inflammatory bowel disease (ssc05321), malaria (ssc05144), intestinal immune network for IgA production (ssc04672) and Chagas disease (ssc05142) were shared among the infected samples at 10, 25, and 50 DPI. Noteworthy, immune-related pathways were enriched at all time points, and all of the significantly enriched KEGG pathways involved in cancer were found at 25 DPI.

### MicroRNA–Gene Network Analysis

**FIGURE 6:**
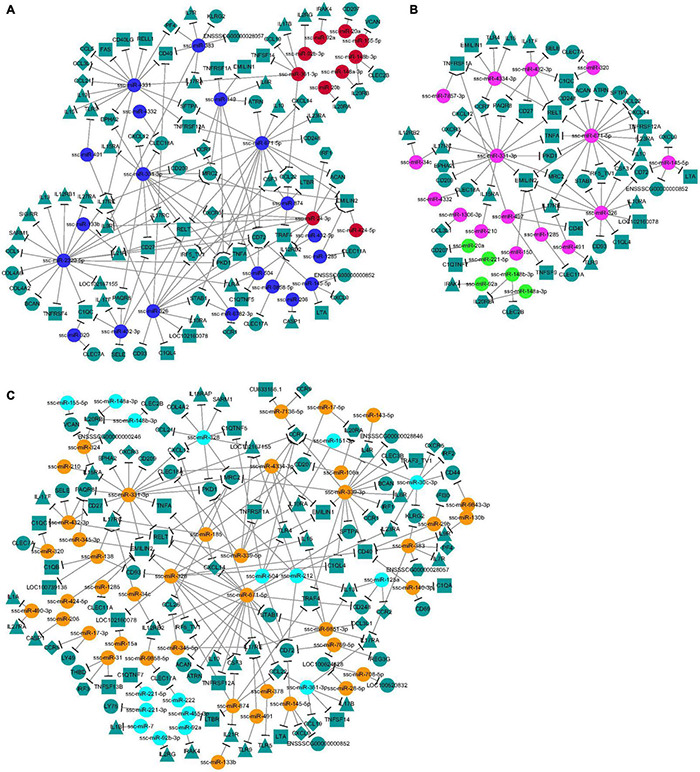
**(A**–**C)** The network analysis of the interaction between the DEMs and immune-related target genes. The node shapes were used for representing the different miRNAs or target genes (different types of cytokines), which were connected by edges (negative interaction between miRNA to target gene). And the colors of spherical nodes were represented the upregulated or downregulated miRNAs in porcine brain at different time points.

Regulatory networks between the immune-related target genes and their upstream regulators (DEMs) at 10, 25, and 50 DPI were constructed to identify crucial connecting nodes (genes or DEMs) (Figure 6). In total, 123 different cytokines, including 19 chemokine related, 30 interleukin (IL) related, 28 tumor necrosis factor (TNF) related, nine interferon (IFN) related, three Toll-like receptors (TLRs), and 34 candidates containing C-type lectin domains were potentially regulated by 71 identified DEMs at the three time points. At 10 DPI, interactions were found between 30 DEMs and 91 cytokines. Among these DEMs, miR-2320-5p regulated the most target genes (20 targets), followed by miR-671-5p (18 targets) and miR-4331 (16 targets); a C-type lectin gene, *CD72*, was regulated by the most DEMs (six DEMs), followed by *EMILIN2*, *IL17RE*, and *MRC2*, each regulated by five DEMs (Figure 6A). These DEMs and cytokines might play important roles in response of the host brain to acute *T. gondii* infection. At 25 DPI, 21 DEMs were found to regulate 55 cytokine genes. Compared with the other DEMs, miR-671-5p potentially participated in the interactions with the most target genes (18 targets) in porcine brain, and TNF-related cytokine *EMILIN2* was regulated by the most DEMs (five DEMs), followed by *IL17RE* and *RELT*, each regulated by four DEMs (Figure 6B). At 50 DPI, 112 different cytokines were regulated by 58 DEMs. Of these, miR-671-5p had the most target genes, with 18 targets, followed by the miRNAs miR-339-3p and miR-331-3p, which each had 15 targets. The target gene regulated by the most DEMs was chemokine-related cytokine *CCR7*, with seven miRNAs. In addition, *CD72*, *EMILIN2*, and *IL17RE* were each regulated by six DEMs (Figure 6C). These cytokines may play key roles in the interactions between porcine brain and *T. gondii* during chronic infection.

### Verification of Differentially Expressed MicroRNAs by RT-*q*PCR

**FIGURE 7:**
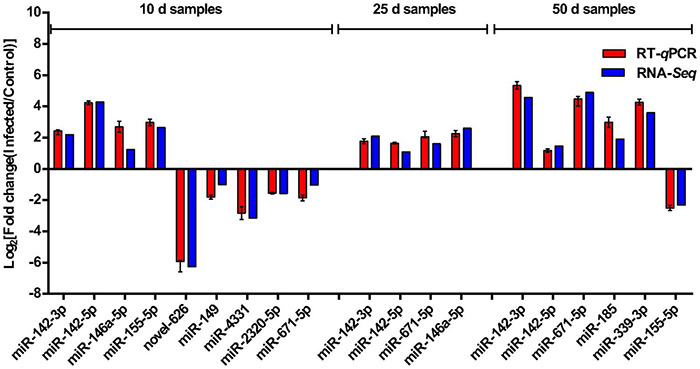
Sequencing data validated by RT-*q*PCR. Comparison of the expression pattern of the sequencing data and RT-*q*PCR data. Log_2_ (fold change) > 0 indicates the transcript upregulated in infection group compared to the control group. Log_2_ (fold change) < 0 indicates the transcript downregulated in infection group compared to the control group.

Eleven representative DEMs, including three miRNAs (miR-142-3p, miR-142-5p, and miR-671-5p) that showed significant differential expression between the infected pigs and control samples at the three time points, were validated by RT-*q*PCR. miRNA sequences were used as the forward primers for the real-time PCR described in Table 4. The expression trends of all the miRNAs measured with RT-*q*PCR corresponded with those obtained from the original small RNA-sequencing data (Figure 7), indicating the accuracy and reliability of sequencing results used for the miRNA expression profiling study.

**TABLE 4:** The sequences of miRNAs used for RT-qPCR validation.

miRNAs	Sequences
miR-142-3p	UGUAGUGUUUCCUACUUUAUGG
miR-142-5p	CAUAAAGUAGAAAGCACUACU
miR-2320-5p	UGGCACAGGGUCCAGCUGUCGG
miR-671-5p	AGGAAGCCCUGGAGGGGCUGGAGG
miR-146a-5p	UGAGAACUGAAUUCCAUGGGUU
miR-155-5p	UUAAUGCUAAUUGUGAUAGGGG
miR-185	UGGAGAGAAAGGCAGUUCCUGA
miR-339-3p	AGCUCCUCGAGGCCAGAGCCC
novel-626	UGGCUCCCUCCACCCGCC
miR-149	UCUGGCUCCGUGUCUUCACUCCC
miR-4331	UGUGGCUGUGGUGUAGGCCAGC

## Discussion

*Toxoplasma gondii* can infect various types of brain cell, including microglia, astrocytes, and neurons. *Toxoplasma*-induced CNS pathology is also common in patients with AIDS (Luft et al., 1984; Luft and Remington, 1988). Bioluminescence imaging alongside confocal fluorescence techniques demonstrate that *Toxoplasma* is localized to the brain, where it is observed predominantly in neurons during chronic infection in mice (Dellacasa-Lindberg et al., 2007; Melzer et al., 2010). However, in addition to mechanical damage caused by the living space of the tissue cysts, the mechanisms by which *T. gondii* injures the host brain remain poorly understood. *T. gondii* has been indicated to have an inherent ability to manipulate the global gene expression profiles of the invaded host cells (Tanaka et al., 2013). Therefore, the potential functions and relationships of target genes regulated by the DEMs identified herein could be used to analyze the possible mechanisms of brain injury during *T. gondii* infection.

### Characterization of Pig Brain MicroRNAs After Infection With *Toxoplasma gondii*

In the present study, a total of 182 DEMs were identified in porcine brain samples at three time points after *T. gondii* infection. Of the 182 DEMs and 102 sample-specific DEMs, 74.18% (135/182) and 64.71% (66/102) were found at 50 DPI, respectively, indicating that chronic *T. gondii* infection induced a high number of DEMs to result in damage to the brain. This result is consistent with those of previous studies (Xu et al., 2013; Hu et al., 2018; Hou et al., 2019; Zhou et al., 2020). According to our results, four (10.26%, 4/39), 12 (24.0%, 12/50), and 43 (91.5%, 43/47) DEMs had consistent expression patterns between 10 and 25 DPI, 10 and 50 DPI, and 25 and 50 DPI, respectively. DEMs with different expression patterns between 10 and 50 DPI might represent the differences in the induced responses of the host between acute and chronic *T. gondii* infection. In addition, the host responses to *T. gondii* infection might be potentiated by similar mechanisms based on the regulation of target gene expression by these DEMs at 25 and 50 DPI. In addition, the expression patterns of numerous DEM homologs, including miR-142-3p, miR-142-5p, miR-146a-5p, miR-155-5p, let-7d-5p, miR-125a, miR-455-3p, and miR-17-5p, obtained from the porcine brain in this study, were similar to those miRNAs identified in mouse brains following acute and chronic *T. gondii* infection (Zhou et al., 2020). Based on these findings, we suggest that these DEMs or DEM homologs induced by *T. gondii* serve as essential mediators in the brain response, but are not strictly host specific. miR-142-3p and miR-142-5p were identified as showing upregulated expression in the brain tissue of pigs following *T. gondii* infection at all time points. miR-142-3p reduces regulatory T cell (Treg) functions by limiting AC9 and cAMP production (Huang et al., 2009), and manages autophagy and thymic-derived regulatory T cell survival by regulating the autophagy gene *ATG16L1* post transcriptionally (Zhai et al., 2014; Lu et al., 2018). miR-142-5p is usually associated with adaptive immune responses of the host in experimental malaria models (Judice et al., 2016). This indicates that miR-142-3p and miR-142-5p, as two members of the mir-142 family, might have central roles in host resistance to *T. gondii*, and could be responsible for the persistent immune activation and inflammatory response in host brain throughout the infection process.

### Unfolded Protein Response

Cells respond to misfolded proteins in endoplasmic reticulum (ER) or mitochondria via the unfolded protein response (UPR), which is an early stress response to external environmental factors and triggers a series of signal transmission processes. Functional loss of ER induces the UPR to activate cell autophagy or apoptosis, thus leads to abnormal nervous system (Hou et al., 2021). Abnormal aggregation of misfolded proteins is one of the most important pathological traits in the brain shared by many neurodegenerative diseases, and there is a significantly causal association between accumulation of a specific misfolded protein and the development of pathology of the brain (Hetz and Saxena, 2017). In the present study, 33 target genes related to the unfolded or misfolded protein response based on 17 GO terms were identified as potential targets of 32 DEMs at three time points (Supplementary Table 8). Of 32 DEMs, miR-92a and miR-92b-3p, circulating biomarkers of *T. gondii* infection (Ngô et al., 2017), were found to be targeted to the gene encoding clusterin (*CLU*). CLU is a secreted chaperone and proposed to be a biosensor of oxidative injury, which helps the protein folding of secreted proteins and participates in proapoptosis and antiapoptosis. CLU-enhanced cell survival can be mediated by activation of UPR/ER stress in hypoxia (Dairi et al., 2016), indicating that *T. gondii*-induced brain injury might be enhanced via potential targeting to *CLU* by the downregulated miR-92a and miR-92b-3p during chronic infection, while neuroprotection is potentially demonstrated based on the upregulated miR-92a and miR-92b-3p during acute infection.

Unfolded protein response controlled by ER stress sensor proteins involves three main proteins: protein kinase RNA-like ER kinase (PERK), inositol-requiring enzyme 1 (IRE1), and activating transcription factor-6 (ATF6). Abnormal expression of ATF6 can promote ER stress-induced inflammation and apoptosis (Chen et al., 2020). In the present study, GO terms associated with PERK- and IRE1-mediated UPR were enriched by DEMs at 10 DPI. In these GO terms, non-receptor-type protein phosphatase 1 (*PTPN1*), mediating AD-like tau pathology in the hippocampus of wild-type mice and synaptic and memory disorders in AD (Hou et al., 2020), was potentially targeted by miR-149, which was downregulated at 10 DPI. *ATF6* was potentially regulated by miR-133a-5p (upregulated at 50 DPI) and miR-4331 (downregulated at 10 DPI), showing the significant difference in the ATF6-induced UPR to ER stress in *T. gondii*-infected brain during the acute and chronic phases of infection. Thus, *T. gondii* might determine the expression of a range of genes encoding overlapping functions to create a dynamic UPR network by regulating brain miRNA expression in the host. UPR signaling pathways have crucial roles in disease pathogenesis, including toxoplasmosis. Simultaneously, perturbation of host mitochondrial function caused by the misfolding protein response following *T. gondii* infection is also likely to impact the pathogenesis of this disease.

### Oxidative Stress

Excessive intracellular amounts of reactive oxygen species (ROS) can lead to oxidative stress (OS). OS and increased ROS production contribute to ER stress, protein misfolding, and induction of the UPR (Zhang and Kaufman, 2006). OS has important roles in toxoplasmic encephalitis-induced neuropathology (Dincel and Atmaca, 2016) and acute toxoplasmosis in mice (Engin et al., 2012; Tonin et al., 2014; Xu et al., 2015). In the current study, 38, 24, and 42 target genes in OS-related GO terms were potentially enriched by DEMs at 10, 25, and 50 DPI, respectively (Supplementary Table 9). Of these, 22 target genes were shared by all three time points. ROS mediates oxidative damage, and is involved in infection-mediated neuropathy (Peterson and Toborek, 2014). *NOX1* can generate ROS, and was potentially targeted by novel-695 and miR-4331 at 10 DPI, and by miR-185 and miR-339-3p at 50 DPI in this study. This implied that augmented OS induced by *NOX1* may contribute to the oxidative damage of cellular membranes and macromolecules, and thus led to cellular apoptosis and death by dysregulation of miRNAs in the acute infection. Approximately 18 genes (*PXDN*, *SESN2*, *ADPRHL2*, *CLN8*, *ERCC3*, *GPX5*, *HYAL2*, *NEIL1*, *NONO*, *NOR*1, *PARK7*, *PDK2*, *PLK3*, *PRDX5*, *PSAP*, *PTGS1*, *ROMO1* and *STX4*) were identified as potential targets by five DEMs (miR-331-3p, miR-326, miR-671-5p, miR-145-5p, and miR-1285), showing decreased expression at 10 DPI, and increased expression at 25 and 50 DPI (Supplementary Table 9). These DEM–target gene networks demonstrated the involvement of OS during the different infection stages, and revealed the possible mechanisms of neuropathological damage induced by the early toxoplasma infection and plerosis in chronic infection phase. Moreover, miR-345-5p, showing upregulated expression in the infected brain at 50 DPI, targets the gene encoding nitric oxide synthase 3 (*NOS3*), which induces nitric oxide (NO) production in endothelial cells and has a neuroprotective effect through maintaining cerebral blood flow. A previous study showed that high NO production, glial activation, and apoptosis cause severe neuropathology in toxoplasmic encephalitis (Dincel and Atmaca, 2016). Thus, miR-345-5p/*NOS3* could reflect a defense mechanism of the host brain against *T. gondii*-induced damage.

### Autophagy

Autophagy is a self-digestive lysosomal degradation pathway found in most eukaryotic cells (Besteiro, 2019), and also has a housekeeping role in removing misfolded or aggregated proteins and clearing damaged organelles (Boya et al., 2013). Tissue-type plasminogen activator can reduce brain injury severity and protected mitochondria via regulation of autophagy (Cai et al., 2021), suggesting that autophagy plays an important role in nervous system injury. Autophagy and beclin1 regulator 1 (*AMBRA1*) is a crucial regulator of autophagy initiation (Di Bartolomeo et al., 2010; Strappazzon et al., 2011). Microtubule-associated protein 1 light chain 3 alpha (*MAP1LC3A*) and its homologs (*GABARAP* and *GABARAPL1*) are a hallmark of autophagy and essential for autophagosome formation (Ye et al., 2020). Previous research had suggested a role for autophagy in the elimination of the *T. gondii* tachyzoites (Ling et al., 2006). In the current study, 47, 29, and 44 target genes were enriched at 10, 25 and 50 DPI, respectively, by the DEMs in GO or KEGG terms tagged with autophagy (Supplementary Table 10). Of these, 24 potential target genes were co-regulated by DEMs across all three time points. *AMBRA1* was potentially targeted by miR-145-5p, miR-331-3p, and miR-671-5p, which showed downregulated expression at 10 DPI and upregulated expression at 25 and 50 DPI (Supplementary Table 10). It indicated that an intracellular defense mechanism is established through initiation of autophagy during acute *T. gondii* infection, whereas autophagy is suppressed during chronic infection. *MAP1LC3A*, *GABARAP* and *GABARAPL1* were identified as the potential targets of miR-324, miR-339-5p, miR-4334-3p, miR-9858-5p, miR-133b, miR-210, and miR-708-5p (Supplementary Table 10). The interactions between targets and miRNAs might be associated with long-term parasitism and chronic injury in the brain during chronic *T. gondii* infection. Frontline studies in neuroscience have revealed that synaptic components (e.g., synaptic proteins, organelles, neurotransmitters, and their receptors) are selectively degraded by autophagy. Apart from the canonical role of autophagy in supporting cell viability, synaptic autophagy appears to regulate synapse remodeling and plasticity (Tomoda et al., 2020). Although autophagy has a crucial role in intracellular defense against *T. gondii*, dysfunctional autophagy might be one of the important mechanisms of pathological injury induced by *T. gondii* infection.

### Cell Apoptosis

Dysregulated cell apoptosis is an important factor that leads to pathology, including developmental defects, autoimmune diseases, neurodegeneration, or cancer (Elmore, 2007). To evade immunity and enhance intracellular survival, *T. gondii* has evolved strategies to modulate host cell apoptosis. The apoptosis of astrocytes in mice is one of the key factors allowing the persistence of this parasite in the brain (Contreras-Ochoa et al., 2013). Previous studies have shown that some miRNAs participate in the regulation of apoptosis in host cells following *T. gondii* infection (Xiao and Rajewsky, 2009; Cai et al., 2013, 2014; Rezaei et al., 2018). In the current study, 249, 155, and 261 potential target genes were enriched in GO and KEGG pathways related to apoptosis by 43, 26 and 71 DEMs at 10, 25 and 50 DPI, respectively (Supplementary Figures 1–3). Of these, 27, 19, and 26 potential target genes were specifically enriched in neuron apoptotic process-related GO terms by 24, 14, and 26 DEMs at 10, 25, and 50 DPI, respectively (Supplementary Table 11), indicating that *T. gondii* is able to alter the neuron apoptotic machinery.

Factor associated suicide (*FAS*) is a key factor that triggers the apoptotic process of host cells. *CJUN* is an important transcription factor that directs the transcription of pro-apoptotic mediators (Chipuk et al., 2010). In the current study, potential targets *FAS*, *CJUN*, *TP63*, *CNTF*, and *TYRO3* were specifically regulated by DEMs (novel-626, miR-2320-5p, miR-4331, miR-2320-5p, miR-6782-3p, novel-511, and miR-149), showing decreased expression at 10 DPI. The interactions between these DEMs and target genes suggest that proapoptotic activity is initiated during acute *T. gondii* infection.

SET nuclear proto-oncogene (*SET*) is often overexpressed in cancer cells, and inhibits p53 activity, which induces cell cycle arrest and apoptosis (Wang et al., 2016). Amyloid precursor protein (*APP*) is closely related to Alzheimer’s disease, and has an important role in apoptosis and pathological injury in the brain (Ułamek-Kozioł et al., 2020). Vascular endothelial growth factor-B (*VEGFB*) is associated with angiogenesis, neuronal protection, and the pathogenesis of tumorigenesis (Dmytriyeva et al., 2020). In the current study, potential targets *PINK1*, *SET*, *APP*, and *VEGFB* were also specifically regulated by DEMs (miR-708-5p, miR-769-5p, miR-31, and miR-708-5p), showing upregulated expression at 50 DPI. These DEMs obtained at 50 DPI might be involved in pathological damage of brain induced by *T. gondii* cysts.

Approximately 37 target genes in the apoptosis pathway (ssc04210) were identified as potential targets by 44 DEMs at three time points (Supplementary Figure 4), of which 21 genes were co-regulated by the DEMs obtained at three time points. All DEMs participating in the regulation of these genes at 10 DPI were identified as having downregulated expression, whereas the expression of miRNAs obtained at 25 DPI and 50 DPI showed the opposite expression pattern. This shows that *T. gondii* alters the apoptosis pathway in host cells during acute and chronic infections. In addition, the hypofunction of miR-149, miR-331-3p and miR-671-5p might activate the TNF receptor pathway of cell apoptosis by inducing increased expression of TNFA and TNFAR1 during acute *T. gondii* infection (Supplementary Table 12). The alterations reduce the survival and proliferation of *T. gondii* and make the parasites susceptible to immune attack, thereby attenuate the acute damage in the brain induced by *T. gondii*.

### Tumorigenesis

Brain tumors often result in space-occupying lesions and compromise the blood-brain barrier, causing a robust infiltration of multiple immune cell types from the peripheral circulation (Quail and Joyce, 2017), further aggravating the degree of brain damage. Brain tumorigenesis can be induced by various factors, including brain injury and intracranial infection. A positive correlation between anti-*T. gondii* antibodies and incidences of brain cancer was shown in the previous studies, which suggested *T. gondii* infections increase the risk of brain cancer (Thomas et al., 2012; Thirugnanam et al., 2013). *T. gondii* infection can cause gliomas in experimental animals (Wrensch et al., 1993). In the present study, all of the significantly enriched KEGG pathways involved in cancer, including acute myeloid leukemia (ssc05221), endometrial cancer (ssc05213), bladder cancer (ssc05219), non-small cell lung cancer (ssc05223), glioma (ssc05214), and proteoglycans in cancer (ssc05205), were found at 25 DPI, illustrating that mechanisms of many different types of tumor are potentially activated in the infected brain by *T. gondii* infection at this time point. In particular, in the glioma pathway, expression of *PRKCG*, *NRAS*, *MAPK1*, *ARAF*, and *MAP2K2* was potentially dominated by miR-671-5p, miR-152, miR-320, miR-491, and miR-326, with upregulated expression at 25 DPI (Supplementary Table 13), suggesting that cell growth and proliferation are influenced by *T. gondii* via the MAPK signaling pathway. Brain injury induced by acute *T. gondii* infection might be suppressed at 25 days post-infection through inhibition of tumorigenesis signaling pathway-related genes expression. Moreover, activity of the mTOR signaling pathway might be inhibited by *Toxoplasma* infection to regulate cell survival based on the control of *AKT2* and *PIK3CD* expression by miR-671-5p and miR-150 (Supplementary Figure 5 and Supplementary Table 13). *AKT2* is one of the important genes associated with signaling pathways that control glial cell activation, which serves as a compensatory response that modulates tissue damage and recovery (Chen et al., 2019). The current study also identified up to 17 DEMs (miR-150, miR-331-3p, miR-671-5p, miR-326, miR-432-3p, miR-491, miR-4332, miR-4334-3p, miR-320, miR-152, miR-34c, miR-497, miR-1306-3p, miR-92a, miR-128, let-7e, and miR-664-3p) participating in cancer processes at 25 DPI. The above analyses indicated that brain injury caused by *T. gondii* may be closely related to the mechanism of brain tumorigenesis. Intriguingly, nearly all these DEMs (exception for let-7e) were also found to have an important role in regulation of apoptosis. This suggests that that *T. gondii* initiates cancer processes through dysregulation of apoptosis induced by these DEMs.

### Inflammatory Response

Neuroinflammation is a physiological protective response in the context of infection and injury, and is believed to play a pivotal role in brain injury (Chang et al., 2020). However, neuroinflammation, especially if chronic, may also drive neurodegeneration (Yilmaz et al., 2019). Activated immune cells during infection produce inflammatory factors that can be cytotoxic and cause brain injury (Quail and Joyce, 2017). Previous research has found that *T. gondii* infection stimulated the neurons and the entire brain to induce various inflammatory diseases, such as subclinical and basal neuroinflammation, chorioretinitis, and encephalitis (Weiss and Dubey, 2009; Parlog et al., 2014), leading to brain injury and cognitive dysfunction (Kang et al., 2020). Many miRNAs involved in the inflammatory response and pathological injury of brain tissue in mice or human; for example, experimental autoimmune encephalomyelitis disease severity was reduced by overexpression of miR-146a, miR-497, miR-26a, and miR-20b, or by suppression of miR-181c and miR-155 (Martinez and Peplow, 2020). In addition, let-7, especially let-7d and let-7e, was previously reported as a signaling molecule for TLR7 in microglia and macrophages, which contribute to CNS inflammation (Lehmann et al., 2012; Derkow et al., 2018). These miRNAs were also identified in the current study, revealing their possible roles in inflammation-induced pathological injury in the brain during *T. gondii* infection.

The gut contains more neural networks and is more closely linked to the nervous system than is commonly perceived. For example, gastrointestinal inflammation has been associated with schizophrenia (Severance et al., 2012, 2016). The gut mucosal immune system has a crucial role in the systemic dissemination of *T. gondii* (Hinze-Selch, 2015). In the KEGG enrichment, two gut mucosal immune-related pathways [inflammatory bowel disease (ssc05321) and intestinal immune network for IgA production (ssc04672)] were shared among the infected brains at 10, 25, and 50 DPI. The significant enrichment of these two immune-related pathways suggests that these immune factors are implicated in brain disorder pathogenesis, and implies a suggested link between the mucosa and brain immunity during *T. gondii* infection.

The NF-kappa B (NF-κB) (ssc04064) and T cell receptor (TCR) (ssc04660) signaling pathways were significantly enriched at both 10 and 50 DPI (Supplementary Table 7). miR-145-5p and miR-31 have been shown to influence the development of inflammation by activating the NF-κB pathway (Shi et al., 2018; Wang X. et al., 2019). miR-145-5p/NF-κB or miR-31/NF-κB was also found to have a possible role in *T. gondii* infection in the current study. *T. gondii* activated or inhibited the NF-κB signaling pathway by regulating various stimulating factors, including LIGHT, LTA, and TNFα (encoded by *TNFSF14*, *LTA*, and *TNFA*, respectively). However, the miRNAs(miR-361-3p, miR-331-3p, miR-671-5p, and miR-145-5p), as potential regulators of the NF-κB signaling pathway, showed the opposite expression patterns in acute and chronic infection stages (Supplementary Table 14). Moreover, stage-specific stimulating factors were also found to be altered; for example, acute infection might activate the NF-κB signaling pathway via CD40 (encoded by *CD40G*), which was regulated by miR-4331 with decreased expression, whereas chronic infection might regulate this pathway through miR-31/TNFSF13B or miR-7/IL1B interactions (Supplementary Table 14). These results indicate that *T. gondii* can alter the expression of miRNAs to regulate the NF-κB signaling pathway through modulation of multiple activators at different stages of infection, which might strengthen the inflammatory response to CNS injury, causing neural apoptosis and decreases in synaptic density in the brain (Zhang et al., 2014). A previous study also found that the NF-κB pathway was activated in the brains of chronically infected mice, and activation of the pathway was induced by excessive cytokine expression, such as of TNF, IL-1β, and TLRs (Courtois and Gilmore, 2006). In addition, key genes of the NF-κB signaling pathway were also regulated to influence the TCR signaling pathway. Recent research found that intracerebral T cells are crucial in the control of *T. gondii* infection and are supported by essential functions from other leukocyte populations (Blanchard et al., 2015). In addition, TCR has a key role in the functioning of T cells and formation of the immunological synapse. The current study found that SHP1 (encoded by PTPN6), acting as a crucial negative regulator of TCR-mediated signaling to check the hyperactivation of immune response associated with the pathway (Brockdorff et al., 1999), might be out of control because of decreased miR-30c-3p, let-7a, and let-7d-5p at 50 DPI (Supplementary Table 15). This might represent a host defense mechanism against inflammatory damage of brain tissue induced by chronic *T. gondii* infection. Furthermore, TNFα and IL-10 were regulated by miR-671-5p and miR-331-3p with opposite expression patterns in acute and chronic infections (Supplementary Table 15), indicating an immune regulatory network in which Th1- and Th2-type cytokines co-participated during *T. gondii* infection.

Further analysis of miRNA–gene interactions between immune-related cytokines and DEMs revealed a complex immune regulatory network and the crucial function nodes (DEMs or genes) induced by *T. gondii* in the different infection stages (Figure 6). At 10 DPI, miR-2320-5p, binding to conserved sequences of the Porcine reproductive and respiratory syndrome virus (PRRSV) genome, and having a role in a PRRS infective model (Li et al., 2015), was found to be involved in regulating 20 potential target genes, suggesting that miR-2320-5p might be the most important regulator of the immune regulatory network during acute infection. CD72, a co-receptor of B cell receptor (BCR) and a member of the C-type lectin superfamily, was identified as the potential key gene node, potentially targeted by the DEMs with a decreased expression, including miR-504, miR-145-5p, miR-326, miR-671-5p, miR-149, and miR-2320-5p at 10 DPI. CD72 positively regulates the transition of pre-B cells to mature B cells, and also has opposing effects on B cell survival in different studies (Baba et al., 2005; Li et al., 2006). CD72 might also promote the production of mature B cells and protective antibodies in response to *T. gondii* in the infected brain during acute infection. A similar mechanism was also found in the BCR signaling pathway regulated by the identified miRNAs from porcine spleen in previous research (Hou et al., 2019). In addition, interaction between CD72 and its ligand Sema4D has been shown to be involved in the upregulation of NO production in microglia (Tsuchihashi et al., 2020). Therefore, CD72 may contribute to severe neuropathology in toxoplasmic encephalitis via inducing high NO production (Dincel and Atmaca, 2016).

At 25 and 50 DPI, miR-671-5p was found to participate in interactions with the most target genes in the porcine brain. *EMILIN2* was identified as a crucial gene node at 25 DPI, which was potentially targeted by DEMs with increased expression, including miR-1285, miR-150, miR-326, miR-331-3p, and miR-671-5p. miR-671-5p was shown to alter functionally in human glioblastoma multiforme and to be involved in the modification of the biopathological profile of this cancer (Barbagallo et al., 2016). *EMILIN2* is expressed in the CNS (Alfonso et al., 2011), and can activate the extrinsic apoptotic pathway through direct binding to death receptors 4 and 5 (LeBlanc and Ashkenazi, 2003). Peripheral monocytes/macrophages infiltrating the brain has been found to participate in CNS disease pathogenesis (Haage et al., 2019). And *EMILIN2* serves as one of the best expression markers for identifying peripheral monocytes/macrophages compared to microglia in brain (Haage et al., 2019). Therefore, interactions between *EMILIN2* and its miRNA regulators might have an important role in regulating the inflammatory response and cell apoptosis pathway, and might represent a repair mechanism for acute brain injury during *T. gondii* infection at 25 DPI due to the upregulation of the miRNA regulators of *EMILIN2*.

At 50 DPI, *CCR7* was found to be the key gene node in the immune regulatory network during chronic infection. CCR7, as a well-established homing receptor for dendritic cells and T cells, was potentially regulated by decreased miR-30c-3p and miR-504, and increased miR-331-3p, miR-339-3p, miR-339-5p, miR-4334-3p, and miR-7136-5p in the current study. CCL19 and CCL21 were regulated by decreased miR-361-3p and miR-328, respectively at this time point, which might lead to increased expression of CCL19 and CCL21 in the infected brain. Interactions of CCL19 and CCL21with their ligands might facilitate priming of immune responses. Previous research demonstrated a significant increase in the expression of *CCL19*, *CCL21*, and *CCR7* in CNS over the course of *T. gondii* infection, and that CCR7^–/–^ mice failed to generate sufficient IFN-γ (Noor et al., 2010). CCR7 might also be an important cytokine activating the Th1-type cellular immunity that is the crucial mechanism for controlling parasite replication and causing inflammatory damage during *T. gondii* infection (Noor et al., 2010; Yang et al., 2018).

Although the DEM or gene nodes with the most interactions were different at different infection stages, the current analysis also revealed the commonality or similarity of immunomodulatory mechanisms based on the miRNA–gene interactions induced by *T. gondii* at different infection stages. For instance, sterile alpha and TIR motif containing 1 (SARM1), CCL21, and C1q and TNF-related 5 (C1QTNF5) were potentially targeted by downregulated DEMs at 10 and 50 DPI (Figure 6), which suggested that similar regulatory mechanisms of immune response existed in the host brain during acute and chronic infection with *T. gondii*. In addition, of the 182 DEMs obtained at all time points, 16 miRNAs pointing to the interleukin 17 receptor (IL17R) family, including IL17RE, IL17RA, and IL17RC, might be key cytokines modulating the immune status of the host in *T. gondii* infection. IL17R is targeted by proinflammatory IL17 cytokines (IL17A-F), has a crucial role in inflammatory responses, and contributes to the pathology of many autoimmune diseases (Wu et al., 2011). Excessive cytokine responses can cause neuronal apoptosis and glial damage, decreasing the neurotrophic support and inducing structural changes (Fabiani et al., 2015), which are an important mechanism of *T. gondii*-induced pathological damage in the infected brain.

## Conclusion

This study showed that *T. gondii* infection induced significant changes in the miRNA expression profiles of porcine brain tissues *in vivo*. In total, 182 miRNAs in the porcine brain displayed differential expression. Thus, *T. gondii* appears to interfere with the global miRNA expression profiles of invaded cells to modulate host cell signaling pathways and cellular processes such as UPR, OS, autophagy, apoptosis, tumorigenesis, and inflammatory responses, to enable the parasite to persist in host CNS cells. These results provide important information for understanding the pathogenic mechanism of brain damage induced by *T. gondii* and could aid the development of therapeutic agents against this parasite.

## Data Availability Statement

The datasets supporting the findings of this article are included within the article. Full details of the sequence data were submitted to GEO public database (http://www.ncbi.nlm.nih.gov/geo/) with the accession number GSE176143. The raw data are available in the NCBI Sequence Read Archive under the accession number SRP322697.

## Ethics Statement

The animal study was reviewed and approved by the Animal Care and Use Committee of the College of Veterinary Medicine, Yangzhou University (Approval ID: SYXK [Su] 2012-0029). Animal care and procedures were handled strictly according to the Animal Ethics Procedures and Guidelines of the People’s Republic of China.

## Author Contributions

JT and ZH conceived and designed the experiments, and wrote the manuscript. ZH, LW, DS, WC, and YZ performed the experiments. ZH, DL, JX, SH, and ZP performed the data analysis. ZP helped in the revising of the manuscript. All authors read and approved the final manuscript.

## Conflict of Interest

The authors declare that the research was conducted in the absence of any commercial or financial relationships that could be construed as a potential conflict of interest.

## Publisher’s Note

All claims expressed in this article are solely those of the authors and do not necessarily represent those of their affiliated organizations, or those of the publisher, the editors and the reviewers. Any product that may be evaluated in this article, or claim that may be made by its manufacturer, is not guaranteed or endorsed by the publisher.

## Footnotes

1
http://www.ensembl.org/Sus_scrofa/Info/Index

2
http://www.mirbase.org/

3
http://www.ncbi.nlm.nih.gov/geo/

## Supplementary Material

Supplementary Figure 1 The network analysis of the interaction between the DEMs and apoptosis-related target genes at 10 DPI. The colors of spherical nodes were represented the upregulated miRNAs or downregulated ones of porcine brain.

Supplementary Figure 2 The network analysis of the interaction between the DEMs and apoptosis-related target genes at 25 DPI.

Supplementary Figure 3 The network analysis of the interaction between the DEMs and apoptosis-related target genes at 50 DPI.

Supplementary Figure 4 Target genes of DEMs enriched in Apoptosis pathway between the infected and control groups at 10, 25, and 50 DPI.

Supplementary Figure 5 Target genes of DEMs enriched in Glioma pathway between the infected and control samples at 25 DPI.

Supplementary Table 1 Top 20 most abundant miRNAs of each sample.

Supplementary Table 2 TPM and read counts of the infection stage-specific known miRNAs in pigs.

Supplementary Table 3 Differentially expressed miRNAs between BI-10 and BC-10 samples.

Supplementary Table 4 Differentially expressed miRNAs between BI-25 and BC-25 samples.

Supplementary Table 5 Differentially expressed miRNAs between BI-50 and BC-50 samples.

Supplementary Table 6 GO terms significantly enriched by target genes of DEMs from the samples at 10, 25, and 50 DPI (*P* < 0.01).

Supplementary Table 7 KEGG pathways significantly enriched by target genes of DEMs from the samples at 10, 25, and 50 DPI (*P* < 0.05).

Supplementary Table 8 Unfolded or misfolded protein response-related target genes regulated by the DEMs at 10, 25, and 50 DPI.

Supplementary Table 9 Oxidative stress-related target genes regulated by the DEMs at 10, 25, and 50 DPI.

Supplementary Table 10 Autophagy-related target genes regulated by the DEMs at 10, 25, and 50 DPI.

Supplementary Table 11 Neuron apoptotic process-related target genes regulated by the DEMs at 10, 25, and 50 DPI.

Supplementary Table 12 Target genes from Apoptosis pathway regulated by the DEMs obtained at 10, 25, and 50 DPI.

Supplementary Table 13 Target genes from Glioma pathway regulated by the DEMs obtained at 25 DPI.

Supplementary Table 14 Target genes from NF-κB signaling pathway regulated by the DEMs obtained at 10 and 50 DPI.

Supplementary Table 15 Target genes in TCR signaling pathway regulated by the DEMs obtained at 10 and 50 DPI.
