# Supplementary figures and images for: Global MicroRNAs Expression Profile Analysis Reveals Possible Regulatory Mechanisms of Brain Injury Induced by Toxoplasma gondii Infection
Source: Front Neurosci. 2022 Mar 10;16:827570. doi: 10.3389/fnins.2022.827570 (PMC8961362; doi:10.3389/fnins.2022.827570)

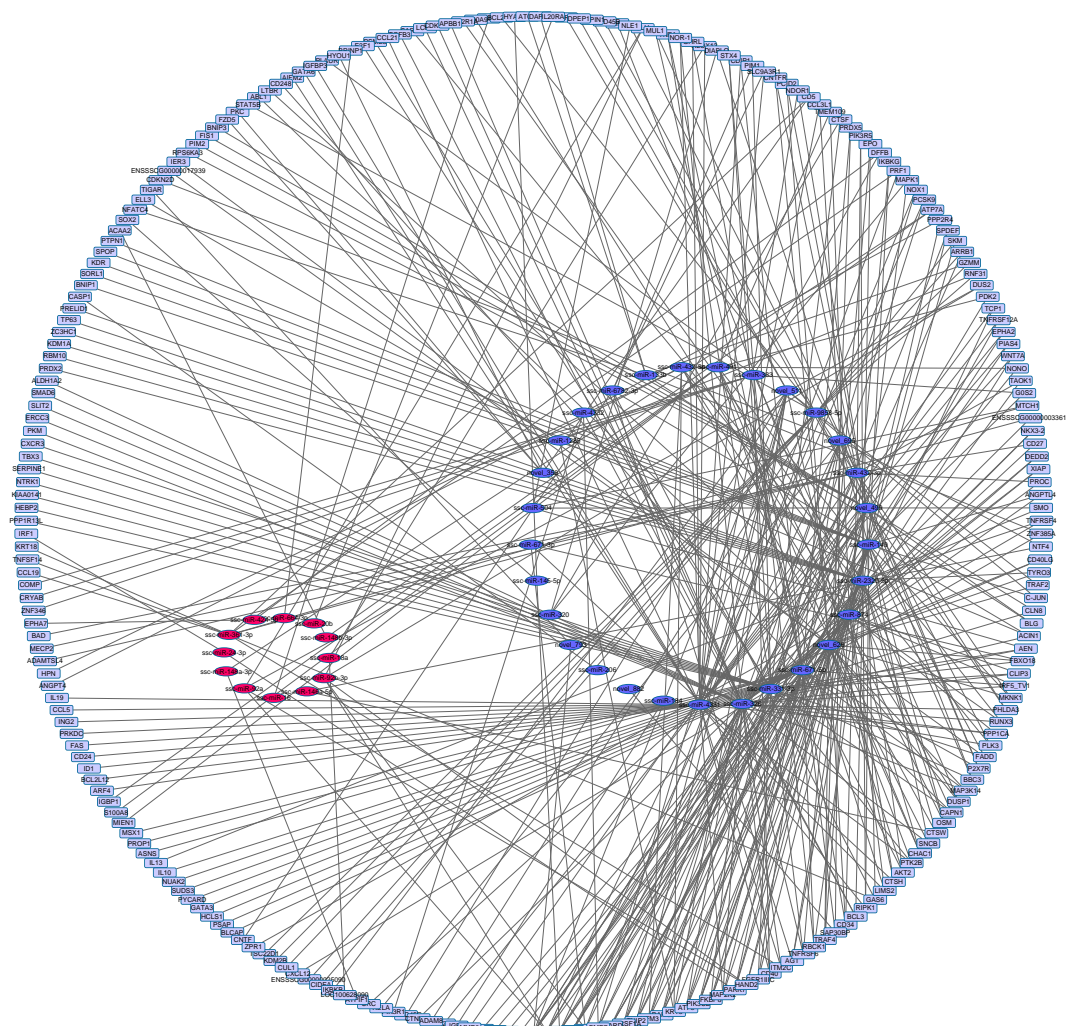

Supplement: Supplementary Figure 1 — The network analysis of the interaction between the DEMs and apoptosis-related target genes at 10 DPI. The colors of spherical nodes were represented the upregulated miRNAs or downregulated ones of porcine brain. [file Image_1.pdf]

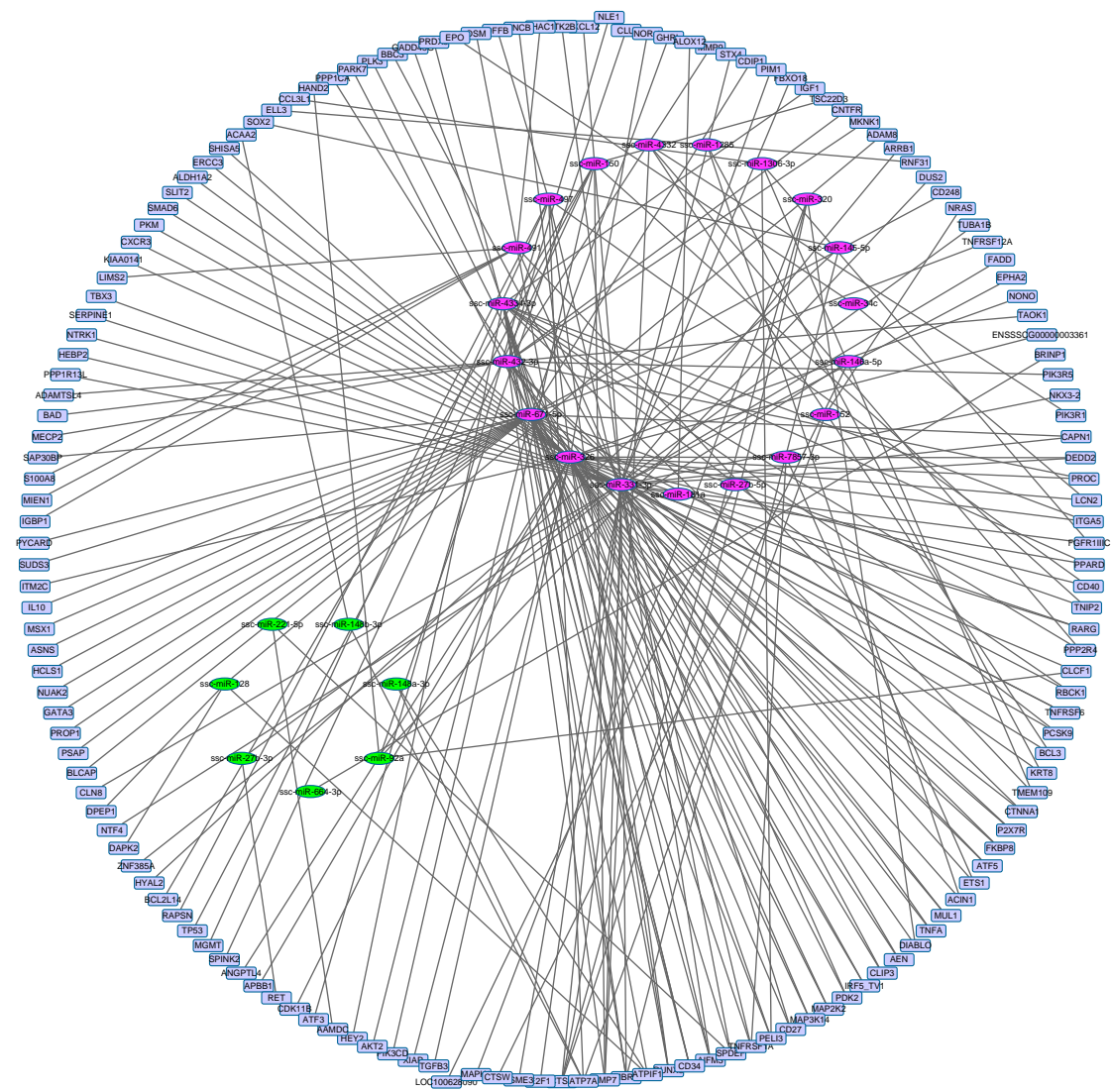

Supplement: Supplementary Figure 2 — The network analysis of the interaction between the DEMs and apoptosis-related target genes at 25 DPI. [file Image_2.pdf]

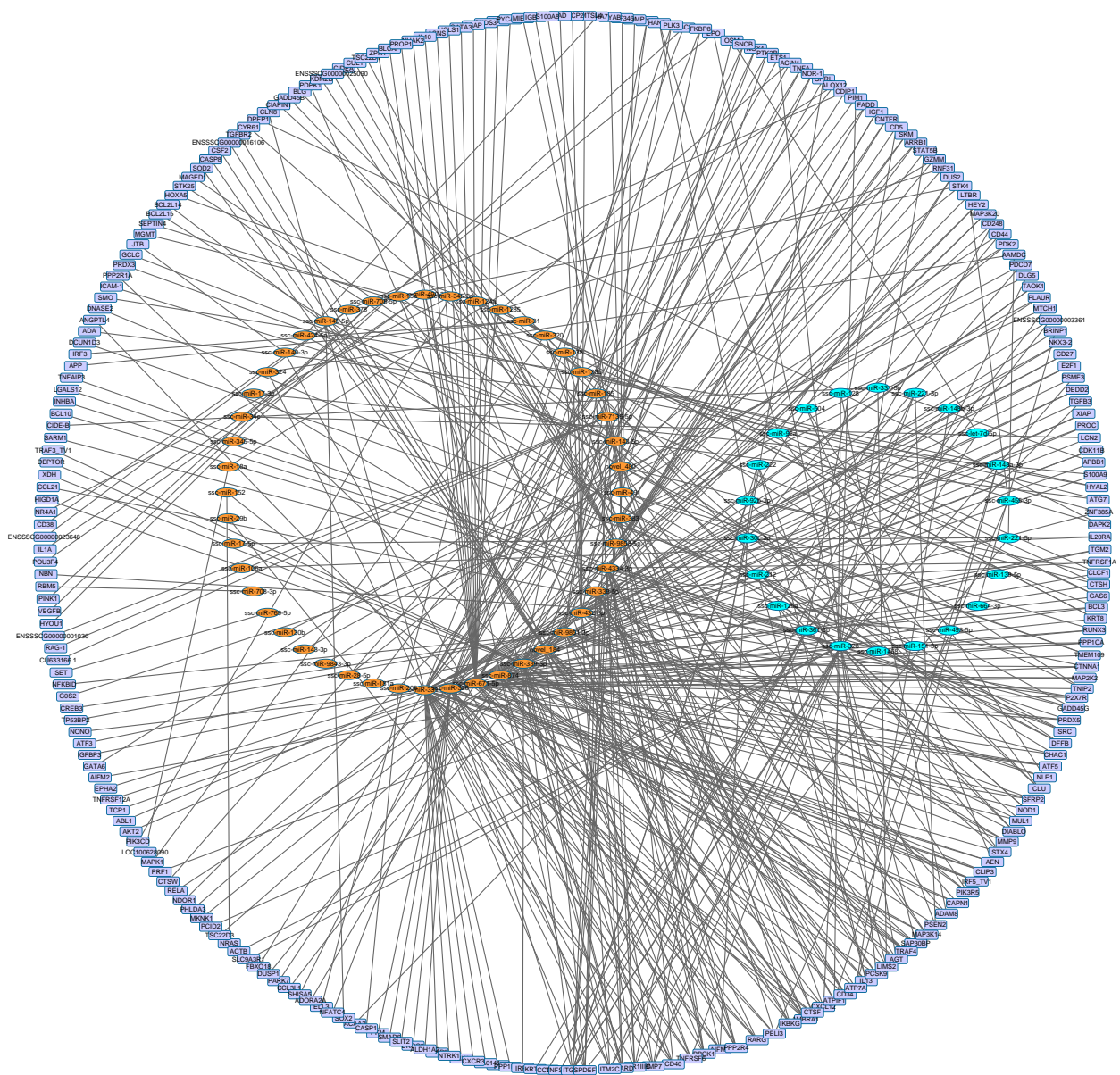

Supplement: Supplementary Figure 3 — The network analysis of the interaction between the DEMs and apoptosis-related target genes at 50 DPI. [file Image_3.pdf]

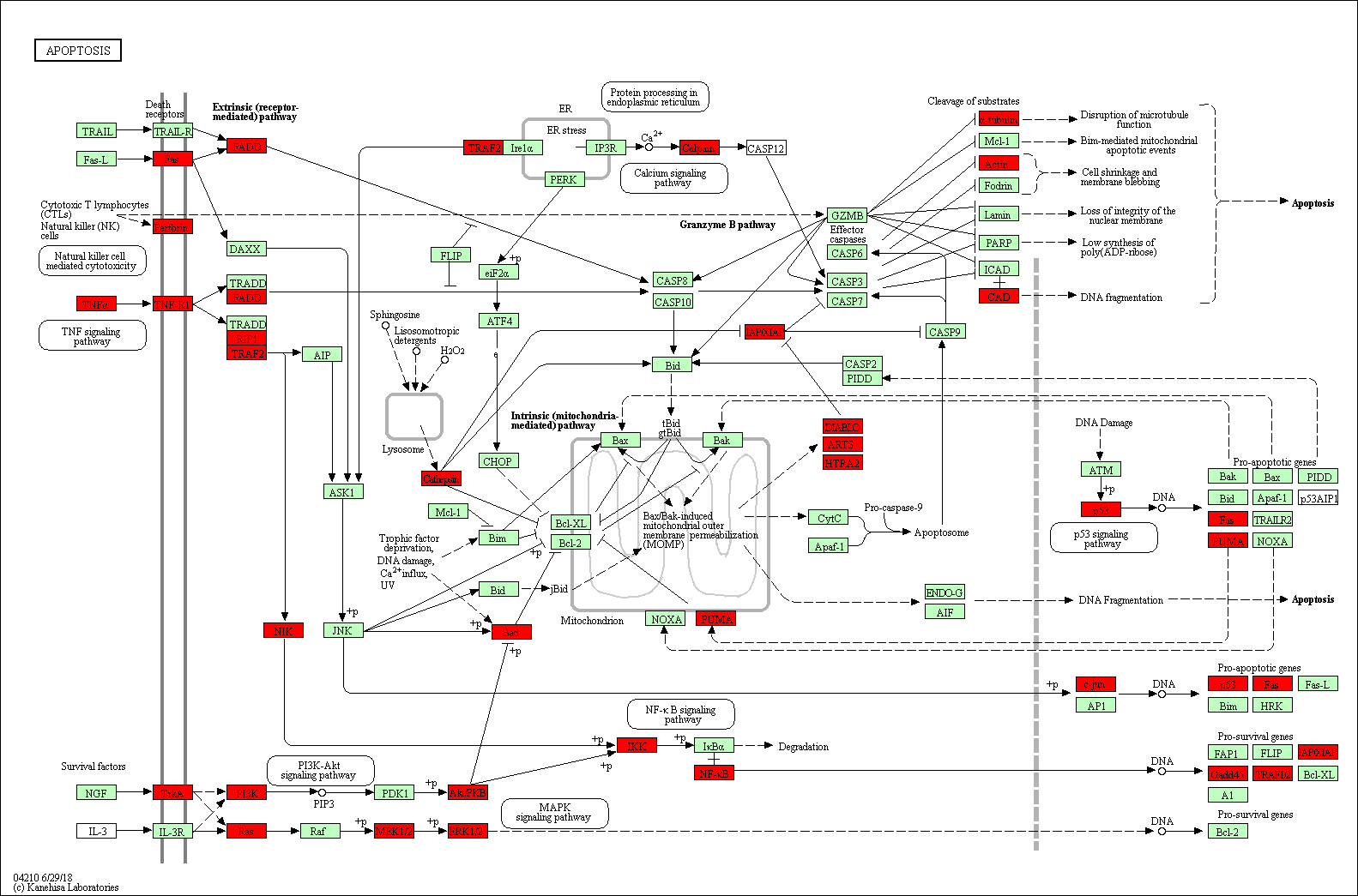

Supplement: Supplementary Figure 4 — Target genes of DEMs enriched in Apoptosis pathway between the infected and control groups at 10, 25, and 50 DPI. [file Image_4.png]

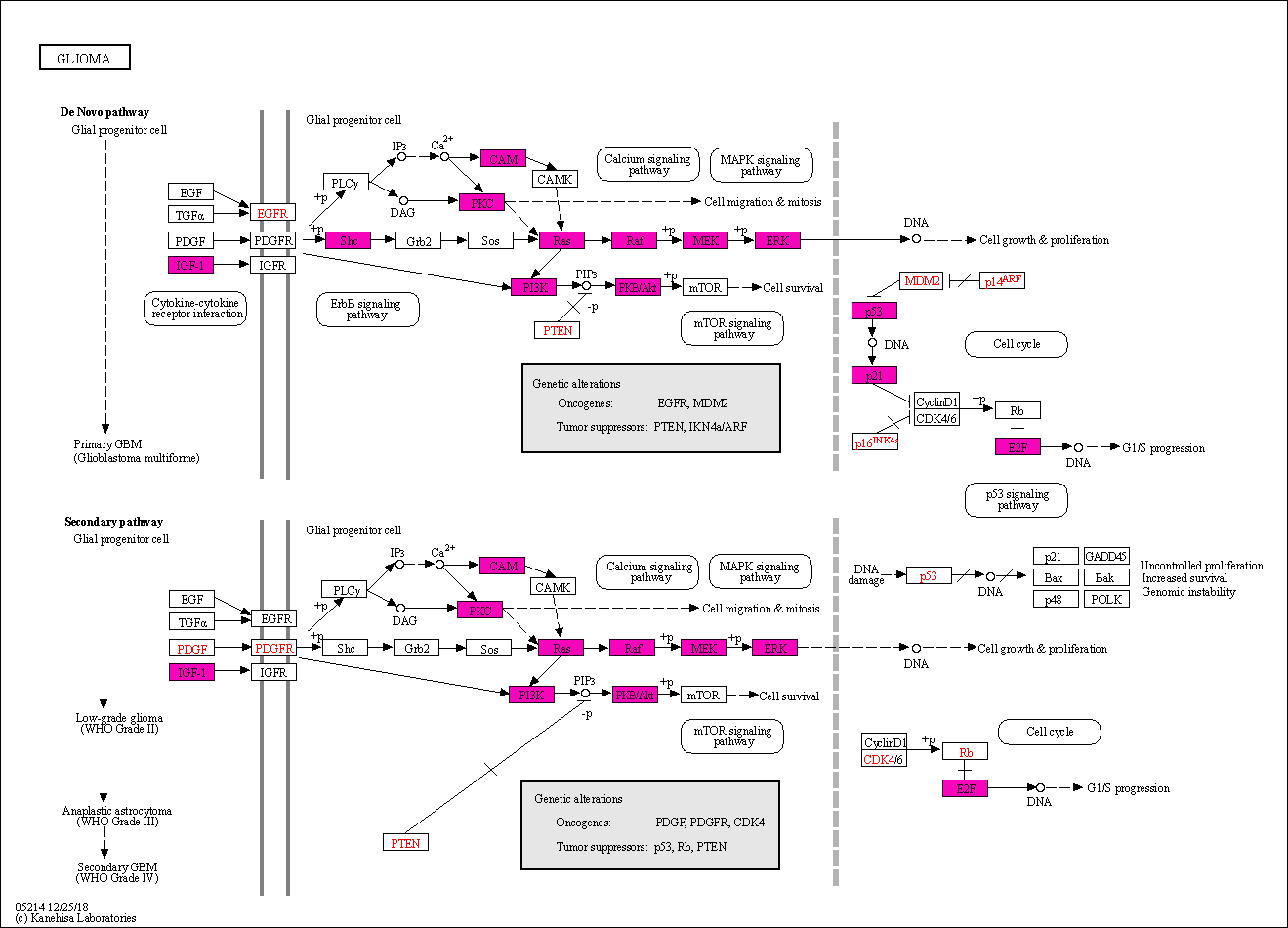

Supplement: Supplementary Figure 5 — Target genes of DEMs enriched in Glioma pathway between the infected and control samples at 25 DPI. [file Image_5.png]
